# Religious-spiritual coping among family members of children requiring continuous and complex care: a mixed-methods study*

**DOI:** 10.1590/1980-220X-REEUSP-2025-0143en

**Published:** 2025-11-28

**Authors:** Bruna Josiane de Lima, Aline Cristiane Cavicchioli Okido, Wendy Sue Looman, Aline Helena Appoloni Eduardo, Fernanda Machado Silva-Rodrigues, Fabrine Aguilar Jardim, Regina Aparecida Garcia Lima

**Affiliations:** 1 Universidade Federal de São Carlos, Programa de Pós-Graduação em Enfermagem, São Carlos, SP, Brazil.; 2 Universidade de São Paulo, Escola de Enfermagem de Ribeirão Preto, Departamento de Enfermagem Materno-Infantil e Saúde Pública, Ribeirão Preto, SP, Brazil.; 3 University of Minnesota Twin Cities, School of Nursing, Minneapolis, United States.; 4 Universidade de São Paulo, Escola de Enfermagem, São Paulo, SP, Brazil.

**Keywords:** Child, Family, Religion, Spirituality, Chronic Disease., Criança, Família, Religião, Espiritualidade, Doença Crônica

## Abstract

**Objective:**

to analyze the use of religious-spiritual coping among family members of children who require continuous and complex care

**Methods:**

a mixed-methods study was developed in Brazil with family members of children requiring continuous and complex care. In the quantitative stage, 51 family members answered a demographics survey and the Brief Scale for Spiritual/Religious Coping. In the qualitative stage, 15 family members answered an open-ended question about the practice of religiosity and spirituality in their daily lives.

**Results:**

family members’ age and number of residents in the household were significantly associated with religious-spiritual coping. Three thematic categories emerged from the statements, revealing an experience marked by trust in God’s designs and relief from suffering provided by faith.

**Conclusion:**

family members in this study used positive religious-spiritual coping strategies more frequently, especially those from smaller families. However, younger family members used negative religious-spiritual coping strategies more frequently.

## RESUMO

**Objetivo:**

analisar o uso do *coping* religioso-espiritual entre familiares de crianças que necessitam de cuidados contínuos e complexos. Métodos: estudo de métodos mistos, desenvolvido no Brasil, com familiares de crianças que necessitam de cuidados contínuos e complexos. Na etapa quantitativa, 51 familiares responderam a um questionário demográfico e à *Brief Scale for Spiritual/Religious Coping*. Na etapa qualitativa, 15 familiares responderam a uma pergunta aberta sobre a prática de religiosidade e espiritualidade em suas vidas cotidianas.

**Resultados:**

a idade dos familiares e o número de moradores no domicílio foram significativamente associados ao *coping* religioso-espiritual. Três categorias temáticas emergiram dos depoimentos, revelando uma experiência marcada pela confiança nos desígnios de Deus e pelo alívio do sofrimento proporcionado pela fé.

**Conclusão:**

os familiares deste estudo utilizaram estratégias de *coping* religioso-espiritual positivas com maior frequência, especialmente aqueles de famílias menores. No entanto, os familiares mais jovens utilizaram estratégias de *coping* religioso-espiritual negativas com maior frequência.

## INTRODUCTION

Children requiring continuous and complex care have severe medical conditions, and functional and intellectual limitations^(1,2)^. This group includes children with congenital anomalies and genetic diseases, prematurity and related complications, and cerebral palsy^(3)^. Life-sustaining technological devices, such as gastrostomy and tracheostomy, are common^(2)^. These children require an exhaustive care routine; consequently, parents and other family members experience extreme emotional, physical, social, and financial burdens^(4)^.

Despite these challenges, family members of children who require continuous and complex care seek resources to adapt to the circumstances. Religiosity and spirituality constitute fundamental elements of comfort and strength to overcome difficulties^(5)^. These terms are closely related but conceptually distinct. Religiosity encompasses beliefs and practices that are often associated with adherence to a religious tradition. Spirituality, on the other hand, is a broader concept that refers to the human relationship with what is considered divine, sacred, or transcendent, involving a search for meaning that is not necessarily tied to institutional religion^(6)^.

The term “positive religious-spiritual coping” refers to using religious or spiritual beliefs, behaviors, or faith to minimize or tolerate suffering^(7)^. Conversely negative religious-spiritual coping refers to using strategies that have harmful or negative repercussions, such as questioning God’s existence, delegating the resolution of the problem to God, and understanding a stressful situation as something punishing^(7)^.

There is emerging evidence that certain religious-spiritual coping strategies are associated with better outcomes for caregivers. In a study conducted with caregivers of children with special healthcare needs, family caregivers with no religious belief had a 2.7 times greater chance for high levels of burden when compared to those who endorsing a religious belief^(8)^. Other research found that caregivers of children with medical complexity believed religion and spirituality to help them cope and informed decisions about medical intervention for their children^(9)^.

According to a systematic review and meta-analysis, nurses have proposed several interventions in this area in recent decades. These interventions have been shown to alleviate suffering, enhance hope, assist patients in preparing for end-of-life issues, reduce depressive symptoms, improve quality of life and well-being, decrease perceived stress, and promote empathy. The authors further argue that a holistic nursing approach should include spiritual interventions, as suffering, pain, and negative experiences are often inherent in health-related problems^(10)^.

Although the role of religiosity and spirituality in healthcare has received growing attention, a critical gap remains in understanding how these dimensions are experienced and utilized by family members of children who require continuous and complex care. Investigating religious-spiritual coping in this context is essential for guiding nursing practice and developing holistic care strategies.

To advance the present investigation, the following research question was proposed: how is religious-spiritual coping used by family members of children who require continuous and complex care, considering the influence of sociodemographic characteristics and their everyday experiences of religiosity and spirituality?

Accordingly, this study aimed to analyze religious-spiritual coping among family members of children who need continuous and complex care.

## METHOD

### Study Design

This is a mixed-method study of the sequential explanatory type^(11)^. This approach is characterized by quantitative data collection and analysis in the first stage, followed by qualitative data collection and analysis in the second stage to complement the knowledge about the phenomenon studied. More weight was given to the quantitative stage and less to qualitative data, for which the QUAN-qual notation was used. The quantitative stage was conducted as an observational, analytical, and cross-sectional study, whereas the qualitative stage followed a descriptive and exploratory design.

The findings were integrated in the end of results section using a joint display or comparative matrix, in which qualitative categories were presented side by side with quantitative results. Furthermore, qualitative insights were used in the discussion section to aid in the interpretation of the quantitative findings.

### Population And Selection Criteria

Participants were family members, over 18 years old, of children aged 0 to 12 years who required continuous and complex care residing in a city in the countryside of the state of São Paulo, Brazil. In this study, a family member was considered as any close relative who lived with the child and had affinity and affection ties (e.g., grandparents, uncles). To determine whether a child required continuous and complex care, the following criterion was adopted: the child had to present at least three care demands—developmental care, medication care, technological care, and/or modified routine care^(12)^. The exclusion criterion was any intellectual limitation that could hinder the completion of the data collection instruments. No sample size calculation was performed for this study. A non-probabilistic convenience sampling strategy was used.

### Data Collection

Data collection occurred between July and November 2022. The first participants were recruited from a list of mothers of children requiring continuous and complex care who had already participated in a previous study developed by the research group. Another strategy adopted was disseminating the research in groups on social networks such as WhatsApp^p®^ and Facebook^®^. When a potential participant expressed interest in participating in the study, a home visit was scheduled for data collection. During the visit, other family members who were present were also invited to participate.

In the first stage (QUAN), family members completed a survey developed by the authors to collect the following socio-demographic data: degree of kinship with the child; age; marital status; education; occupation; family income; and the number of residents in the household. A primary caregiver (usually the mother) also answered questions regarding children who need continuous and complex care, such as age and medical diagnosis, in order to more fully characterize the sample.

Subsequently, family members answered the Brief Scale for Spiritual/Religious Coping (SRCOPE), initially developed in the United States and translated and validated into Brazilian Portuguese^(7,13)^. This scale consists of 49 items subdivided into 11 factors, seven related to positive strategies and four to negative strategies. This measure allows the calculation of four indexes: positive SRCOPE - a mean of 34 items related to the positive dimension; negative SRCOPE - a mean of 15 items related to the negative dimension; negative SRCOPE/positive SRCOPE ratio - percentage obtained when calculating the ratio between negative SRCOPE and positive SRCOPE; and total SRCOPE - a mean of the sum between inverted positive SRCOPE and negative SRCOPE.

The resulting values range from 1 to 5, where higher values reflect greater the use of the strategy (positive, negative, and total SRCOPE), except for the ratio index that ranges from 0.2 to 5, where lower values indicate greater use of positive SRCOPE as opposed to negative SRCOPE. Factorial, internal consistency, and correlation analysis indicated the Brazilian SRCOPE Scale as a valid and reliable instrument. The Brazilian version showed an excellent internal consistency level, and Cronbach’s alpha was 0.98 for the positive SRCOPE dimension and 0.86 for the negative SRCOPE dimension^(13)^.

In the second stage, the same inclusion criteria were applied, and all eligible family members were invited to answer the following open-ended question: how are spirituality and religion present in your daily life? Of the 51 family members who participated in the first stage, 15 family members were willing to report their experience in the second stage. The approximate collection time was 20 minutes per participant.

### Data Analysis and Treatment

For the QUAN stage analysis, the four indexes that could be calculated by the SRCOPE, as described above, were considered dependent variables. Independent variables were those obtained from the application of the socio-demographic instrument, which was subdivided into categorical variables (degree of kinship, religion, education, occupation, and marital status) and continuous variables (family member’s age, family income, and the number of residents in the household). Statistical analyses were performed in the Statistical Analysis System version 9.2 for Windows. Simple and multiple linear regression analysis with stepwise selection criteria was used^(14)^. Differences among variables were considered statistically significant when p < 0.05.

In the second stage of analysis, the interviews were transcribed and examined using an inductive content analysis method^(15)^, following the pre-analysis, material exploration, treatment of results, inference, and interpretation phases. Two researchers performed data coding manually, and a third researcher was consulted to resolve any discrepancies prior to the final reading and coding process. From the analysis, three thematic categories emerged: i) “Believing in God strengthens me”: relief from suffering through faith in God; ii) Transformation in ways of thinking, feeling, and interacting with others and oneself; iii) Gratitude to God and trust in divine purposes.

Chart 1 summarizes how the thematic categories were developed based on participant comments, grouped through initial and intermediate codes.

**Chart 1:** Summary of the analytical process of qualitative data ” São Carlos, SP, Brazil, 2022.

Initial codes	Intermediate codes	Thematic categories
*Believing in a God strengthens me.* *It renews our strength every day.* *God is our refuge and our fortress, always.*	Faith helps to face daily problems	
*God represents everything in our lives.* *Without God and Jesus Christ, I am nothing. Without Him, I would not be here today.*	God at the center	
*Mentalizing* *Heartfelt clamoring.* *Praying.* *Prayers and supplications.*	Strategy to connect with God and access spirituality	“Believing in a God strengthens me”: relief from suffering through faith in God
*There are moments when we think we are alone and that we will not succeed* *I cry like every human being.* *There are days when we are down.* *I got weak.* *It made me angry.*	Moments of weakness	
*The child came to improve me as a person.* *I would never be the person I am today if God had not given them to me. I try to police myself to be better*	Reflection of their attitudes	
*Loving your neighbor, exercising charity,* *Inside an NGO helping mothers* *Helping people with psychological support, giving attention and care,*	Offering to help another	Transformation in ways of thinking, feeling, and interacting with others and oneself
*I am not a church person; I created my church inside my house.* *I don’t cling to any religion; I cling to God.* *God, Jesus is above any religion.* *I am not a 100% religious person.* *I stopped going to churches, but I have much faith in God.*	Giving new meaning to religion and/or religious institutions
*I am thankful God has trusted me to care for, love, and protect them.* *Everything has a purpose.* *I trust in Him.*	Gratitude to God	
*If God wants to take them, I will accept it.* *If they had already accomplished his mission, they could leave.* *They no longer needed to suffer.* *This will one day come to an end.*	Acceptance of terminality	Gratitude to God and trust in divine purposes
*Believing in miracles.* *They will be a totally normal child.* *For God, nothing is impossible.*	Hope for improvement	

### Ethical Aspects

This study complied with regulations regarding research with human subjects outlined in Resolution 510/16 of the Brazilian National Health Council. The project was approved by the Research Ethics Committee (Process 5,507,466, dated July 04, 2022). To preserve anonymity, participants in the qualitative phase were identified through alphanumeric coding, based on the chronological order of their participation, as follows: P1, P2, and so on. Participants who agreed to participate in the study were informed and signed the Informed Consent Form.

## RESULTS

### First Stage (Quan)

In the first stage (QUAN), 51 family members of 28 children requiring continuous and complex care participated. Participants included 28 mothers, eight uncles, seven grandparents, five fathers, two stepfathers, and one sister. Family members’ mean age was 40.3 years, ranging from 18 to 77 years. Most participants (80.4%) reported having a partner. Concerning occupation and education, 32 (62.8%) were employed, and 27 (52.9%) had completed high school. The mean family income was R$3,680.00 (*reais*, Brazil’s currency). The mean *per capita* family income was R$1,001.00, ranging from R$166.00 to R$4,000.00 per person. On average, households included 3.7 residents, with a minimum of two and a maximum of six. In terms of religion, evangelical Christians were the most represented group (48.8%), followed by Catholics.

As for children requiring continuous and complex care, the mean age was 5.8 years, equally divided by gender. As for diagnosis, 14 (50%) children had neurological disorders; nine (32%) had severe sequels due to prematurity; and five (18%) had genetic syndromes and/or congenital anomalies.

Table 1 presents the mean, standard deviation, minimum, maximum, median, and quartile values of the four indices and 11 factors calculated by the SRCOPE. This analysis identified that family members had more significant use of positive strategies than negative ones, with the mean negative SRCOPE/positive SRCOPE ratio remaining at 0.56. Family members used the positive factor “positive position before God” more frequently, reaching an average of 4.58. However, the positive factor “actions in search of spiritual help” had the lowest average among family members in this study (2.35).

**Table 1:** Religious-spiritual coping of family members of children who need continuous and complex care (n = 51) according to the mean, standard deviation, minimum value, maximum value, median, and quartiles. São Carlos, SP, Brazil, 2022.

Variables	Mean	SD	Min.	Q1	Median	Q3	Max.
Indices	
Positive SRCOPE	3.28	0.51	2.03	3.03	3.32	3.65	4.00
Negative SRCOPE	1.82	0.63	1.00	1.40	1.73	2.07	3.93
Total SRCOPE	3.73	0.40	2.75	3.51	3.83	3.96	4.49
Negative SRCOPE/positive SRCOPE ratio	0.56	0.22	0.25	0.43	0.50	0.61	1.23
Positive factors	
Factor P1 - Transformation of oneself and/or one’s life	3.52	0.79	1.77	3.16	3.67	3.94	4.88
Factor P2 - Actions in search of spiritual help	2.35	0.98	1.0	1.8	2.4	2.8	4.8
Factor P3 - Offering to help another	3.46	0.93	1.4	3.0	3.4	4.2	5.0
Factor P4 - Positive position before God	4.58	0.61	1.8	4.3	4.8	5.0	5.0
Factor P5 - Search for support from Clergy	3.19	0.89	1.0	2.75	3.25	4.0	4.75
Factor P6 - Active surrender through God/religion/spirituality	4.08	0.90	1.67	3.67	4.33	5.0	5.0
Factor P7 - Quest for spiritual knowledge	2.98	0.93	1.0	2.33	3.0	3.66	5.0
Negative factors	
Factor N1 - Negative reappraisal of God	1.52	0.85	1.0	1.0	1.0	1.8	5.0
Factor N2 - Negative position before God	2.55	0.99	1.0	2.0	2.33	3.0	5.0
Factor N3 - Dissatisfaction with Clergy	1.47	0.95	1.0	1.0	1.0	1.37	4.75
Factor N4 - Negative reappraisal of meaning	2.00	1.05	1.0	1.0	1.67	2.66	4.67

Legend: SD - standard deviation; Min. - minimum value; Max. - maximum value.

Table 2 shows the effect of socio-demographic variables on religious-spiritual coping of family members of children requiring continuous and complex care according to the simple linear regression model. A statistically significant negative association was found between negative SRCOPE and variables such as family members’ age and family income. The higher the age and income, the lower the adoption of negative strategies. Another negative association was identified between the number of residents in the household, positive SRCOPE and total SRCOPE indexes, indicating that individuals in households with fewer people had higher scores for positive strategies. No significant relationships were identified among categorical variables.

**Table 2:** Effect of socio-demographic variables on religious-spiritual coping of family members of children who need continuous and complex care (n = 51), according to the simple linear regression model. São Carlos, SP, Brazil, 2022.

		Positive SRCOPE			Negative SRCOPE			Total SRCOPE			Negative SRCOPE/positive SRCOPE ratio		
Variables	Categories	Beta (SE)	p	R^2^	Beta (SE)	p	R^1^	Beta (SE)	p	R^2^	Beta (SE)	p	R^2^
Kinship	Mother (ref.) Other family members	– -5.27 (4.16)	0.211	0.0317	– -7.17 (4.09)	0.086	0.0589	– 2.38 (4.21)	0.575	0.0065	– -4.04 (4.19)	0.339	0.0186
Family’s age	Continuous variable	0.06 (0.14)	0.674	0.0036	-0.34 (0.13)	**0.014**	0.1177	0.33 (0.14)	**0.019**	0.1065	-0.33 (0.13)	**0.018**	0.1095
Religion	Catholic (ref.) Evangelical Other	– -4.53 (6.11) -6.46 (6.71)	0.463 0.342	0.0246	– 4.86 (6.14) 3.44 (6.74)	0.433 0.612	0.0163	– -6.21 (5.86) -9.19 (6.44)	0.296 0.162	0.0520	– 6.79 (6.00) 7.46 (6.59)	0.265 0.265	0.0398
Education	Elementary school (ref.) High school Higher education	– -6.66 (7.28) -2.35 (7.51)	0.365 0.756	0.0285	– 2.67 (7.23) -3.53 (7.47)	0.713 0.638	0.0390	– -5.81 (7.23) 0.21 (7.47)	0.425 0.978	0.0412	– 5.46 (7.20) -1.31 (7.44)	0.453 0.861	0.0491
Marital status	With partner (ref.) No partner	– -1.87 (5.29)	0.726	0.0025	– -1.68 (5.28)	0.752	0.0021	– 1.18 (5.29)	0.824	0.0010	– -1.62 (5.29)	0.761	0.0019
Occupation	Selfemployed (ref.) Signed contract Household/wage earner Unemployed	– -6.45 (5.55) 0.51 (5.84) 0.71 (9.72)	0.251 0.930 0.942	0.0520	– -0.72 (5.49) -1.44 (5.78) 15.85 (9.61)	0.896 0.804 0.106	0.0722	– -3.51 (5.46) 2.95 (5.75) -14.36 (9.56)	0.524 0.610 0.140	0.0834	– 4.16 (5.33) -3.40 (5.61) 18.39 (9.33)	0.440 0.548 0.055	0.1276
Family income	Continuous variable	-0.08 (0.14)	0.573	0.0065	-0.29 (0.14)	**0.037**	0.0860	0.19 (0.14)	0.182	0.0361	-0.24 (0.14)	0.094	0.0563
Residents in the household	Continuous variable	-0.44 (0.15)	**0.006**	0.1581	0.30 (0.15)	0.056	0.0788	-0.51 (0.15)	**0.001**	0.2088	0.42 (0.15)	**0.008**	0.1460

Legend: beta - the value of the estimate or angular coefficient (slope) in the regression line; SE - standard error of beta; R^2^ - coefficient of determination (% of the variability of the response variable explained by the independent variable). Variables without normal distribution were transformed into ranks.

Finally, statistically significant variables were entered into a multiple linear regression model by backward stepwise (Wald) logistic regression, as shown in Table 3. The relationships between “family age” and negative SRCOPE, and between “residents in the household” and positive SRCOPE, total SRCOPE, and negative SRCOPE/positive SRCOPE ratio remained in the model with statistical significance. In summary, younger family members obtained higher scores for negative SRCOPE, and those who said they lived with fewer people had better indexes for positive religious-spiritual coping strategies.

**Table 3:** Effect of socio-demographic variables on religious-spiritual coping of family members of children who need continuous and complex care (n = 51) according to multiple linear regression model. São Carlos, SP, Brazil, 2022.

Variables		Beta (SE)	p	Partial R^2^
Positive SRCOPE	Residents in the household	-0.44 (0.15)	**0.006**	0.1581
Negative SRCOPE	Family’s age	-0.34 (0.13)	**0.014**	0.1177
Total SRCOPE	Residents in the household	-0.51 (0.15)	**0.001**	0.2088
Negative SRCOPE/positive SRCOPE ratio	Residents in the household	0.42 (0.15)	**0.008**	0.1460

Legend: beta - the value of the estimate or angular coefficient (slope) on the regression line; SE - standard error of beta; R^2^ - coefficient of determination, stepwise criterion of variable selection; partial R^2^ - 0.1581; intercept (SE) - 37.05 (4.10); P < 0.001. Variables without normal distribution were transformed into ranks.

### Second Stage (Qual)

A total of 11 mothers, one grandmother, one aunt, one father, and one uncle participated in the second stage, totaling 15 family members of 13 children who need continuous and complex care. Their testimonies allowed the construction of three thematic categories, which are presented below.

*“Believing in God strengthens me”: relief from suffering through faith in God*

When telling their experiences, families whose comments were included in the first thematic category emphasized the belief in a higher force translated as “faith” that helped them face “difficult moments” such as the impact of diagnosis.

*In difficult moments, for instance, the diagnosis, which was a moment that shook me a lot, I sought faith, I sought strength in God* [...] *because believing in God strengthens me, makes me confident, makes me believe that despite everything He is with me.* (P8, mother of a 10-year-old child with severe autism spectrum disorder)

*When I face difficult moments in life, I catch myself praying; I say my prayers and ask them to strengthen me and help me face that situation.* (P14, mother of a 6-year-old child with cerebral palsy)

The moment of discovering a child’s condition is often permeated by negative feelings towards oneself, such as guilt, revolt, helplessness, and grief, which are feelings classified as negative religious-spiritual coping. However, even in intense fragility, these family members described the importance of “faith in God” to obtain strength and carry on.

*Right from the moment he was born, I felt something was wrong, but I couldn’t explain what was happening. I felt weak a lot; I thought that everything had been caused by a “culprit”, that everything was wrong, and when I saw him, something made me revolt, I had no strength. Today, I say that my faith was lacking.* (P1, grandmother of an 8-year-old child with cerebral palsy)

*There are moments when we even think we are alone and won’t succeed. Sometimes, I even cry like every human being, but then again, I start to mentalize, strengthen myself, and believe in this greater being, our God.* (P14, mother of a 6-year-old child with cerebral palsy)

As the demands placed on children who require continuous and complex care are permanently challenging, faith was also portrayed as a foundation to face daily routine.

*Everything depends on our faith to help us calm down because the day-to-day is challenging with these children.* (P3, mother of a 9-old-year child with severe autism spectrum disorder)

*It is faith that brings us hope for tomorrow* [...] *faith is a foundation; it is the basis, it is the foundation of my life; without it, I could not face the daily problems.* (P7, aunt of a 5-old-year child with severe autism spectrum disorder)

*God is always present in my daily life; I have a lot of faith and always think positively. There are days that we are down, you know, we are human beings, but the next day I get up and believe that God gave me a new opportunity to breathe, so when we are grateful for the little things, God is present in everything.* (P5, mother of a 6-year-old child with severe autism spectrum disorder)

*My faith in the Lord Jesus is what keeps me going. Everything is in the hands of the Lord. Faith takes us far beyond what we can imagine. I believe it renews our strength every day.* (P15, mother of a 7-year-old child with cerebral palsy)

*Believing in God, with faith, I wake up every day. God represents everything in our lives.* (P11, grandfather of a 4-old-year child with cerebral palsy)

The following are examples of strategies for connecting with God and/or accessing spirituality described by family members:

*I already wake up in the morning and say a prayer, thanking God for the night and thanking Him for my health.* (P4, mother of a 3-year-old child with severe autism spectrum disorder)

*Every day I read the Bible, I stop to pray, and I try to be connected. I try to follow this line* [...] of *course we fail, I am human, but this is my purpose, my direction.* (P9, father of a 10-year-old child with severe autism spectrum disorder)

*Whenever I need Him, He is in my life. Yes, I just cry out from my heart, and He always helps me.* (P13, mother of a 7-year-old child with cerebral palsy)

*Transformation in ways of thinking, feeling, and interacting with others and oneself*

Some family members interpreted the experience of caring for a child who needs continuous and complex care as a possibility for spiritual growth and personal transformation.

*I think I would never be the person I am today if God had not given me him.* (P1, grandmother of an 8-year-old child with cerebral palsy)

*My daughter is my divine miracle. She has been reborn again, and I have learned a lot from the challenges I have been facing daily.* (P12, mother of a 5-year-old child with Guillain-Barré syndrome)

Family members also shared examples of transformations in how they act through charitable practices, whether through prayers, emotional support, or even material support to those in need.

*I see that everything has a purpose. If I did not have to go through everything I am going through, I would never be inside an NGO helping other mothers.* (P1, grandmother of an 8-year-old child with cerebral palsy)

*I try to apply the Gospel of Jesus in my life, loving my neighbor, exercising charity, being better today than I was yesterday, and trying to police myself to be better.* (P6, mother of a 10-year-old child with cerebral palsy)

*Within religion and spirituality, I try to help people and listen to the people who need it; this is how I go on, and I get the strength to play.* (P14, mother of a 6-year-old child with cerebral palsy)

*I say my prayers and devotions; I ask for myself and my family, friends, and neighbors. I think that mothers should unite and help each other to help their children. If you find any mother who wants to talk, you can give them my number.* (P3, mother of a 9-old-year child with severe autism spectrum disorder)

The exhausting demand of care required by these children and difficulties related to mobility from one place to another affected how family members relate to religious institutions.

*I believe a lot in the Catholic Church, but with him especially, I created my church inside my house. My opinion is that God is everywhere.* (P1, grandmother of an 8-year-old child with cerebral palsy)

*I was always a student of the Jehovah’s Witness Bible, but after my daughter was born, I stopped studying, I stopped going to churches. I stopped everything, but I have much faith in God, even if I don’t go to church, even if I don’t attend church, I ask God deep down inside me, and that’s it*. (P13, mother of a 7-year-old child with cerebral palsy)

The reports below demonstrate ow some family members value the “spirituality” dimension over the “religiosity” dimension:

*People cling to God; I do not cling to any religion; I cling to God.* (P2, mother of an 8-month-old baby with cerebral palsy)

*For me, God, Jesus, is above any religion or church sign. When you have faith, and He is in your heart, He will walk with you wherever you are.* (P5, mother of a 6-year-old child with severe autism spectrum disorder)

*I am not 100% religious, but I have a lot of faith and believe in a supreme being, which is God. I believe in Mary, our mother, and I also believe a lot in spirituality.* (P14, mother of a 6-year-old child with cerebral palsy)

*Gratitude to God and trust in divine purposes*

Faith in God and trust in divine purposes have helped family members build their resources for coping with the experience of having a child who needs continuous and complex care.

*So, since I was a little girl, I always believed that there was a God who loved and cared for me and that whatever situation happened in my life, whether good or bad, it was because he allowed it and He would be by my side.* (P8, mother of a 10-year-old child with severe autism spectrum disorder)

*He knows that I put myself in His hand and that His will be done. This helps me, gives me a little strength, you know, because I also know that I can count on God.* (P13, mother of a 7-year-old child with cerebral palsy)

Some family members explained how the belief that nothing is impossible for God strengthens their feelings of hope regarding a child’s clinical improvement or ultimate recovery.

*God willing, soon we will be discharged, soon He will take all this off* [referring to the devices such as a catheter, oxygen, for instance]. *Soon he will be a totally normal child, God willing, and I hope this is very close to what I imagine, that’s all I hope.* (P2, mother of an 8-month-old baby with cerebral palsy)

*She always fought for life, so I believe that our faith and her strength to survive, her will to survive, makes us overcome all the challenges. Our faith makes us believe in miracles, that at some point she can overcome and show significant improvement and evolve a lot.* (P10, mother of a 4-year-old child with cerebral palsy)

Furthermore, even when faced with adversities and challenges that permeate these families’ and children’s daily lives, they expressed gratitude to God for their achievements.

*I am thankful every day when I wake up when I see that he is awake; I have made my day. It does not matter if there is nothing to eat or not, whatever happens during the day, as long as he wakes up.* (P1, grandmother of an 8-year-old child with cerebral palsy)

*Today I have only things to be grateful. He is gaining weight; he is interacting; and he is doing well. I think all I have to do for the rest of my life is be thankful for Him.* (P2, mother of an 8-month-old baby with cerebral palsy)

*I am grateful because everything I am achieving for the benefit of my son. I think God is helping me. God is guiding me.* (P3, mother of a 9-old-year child with severe autism spectrum disorder)

*Always in my prayers, I thank God for having granted me the honor of being a mother because I had three miscarriages and underwent many treatments to be able to have my son.* (P6, mother of a 10-year-old child with cerebral palsy)

Finally, some family members described how accepting terminality is also permeated by a belief in God’s purposes. As these participants noted, this is motivated primarily by a desire to end physical suffering usually experienced by children who need continuous and complex care.

*Sometimes people say that I am cold when I say that if God wants to take him away, I will accept it because I do not accept suffering; I do not want to see him suffer. If I have to see him suffer, I accept his departure. The pain of loss will come, but suffering is worse.* (P1, grandmother of an 8-year-old child with cerebral palsy)

*I have also asked God if he had already fulfilled his mission; he could leave so he would not have to suffer anymore.* (P2, mother of an 8-month-old baby with cerebral palsy)

Finally, the Chart 2 below presents a comparative matrix, displaying qualitative findings alongside quantitative results.

**Table:** 

Quantitative findings	Qualitative findings	Interpretation
Mean of positive SRCOPE: 3.28 Mean of negative SRCOPE: 1.82 Ratio: 0.56	Family members described a greater use of positive strategies of religious-spiritual coping versus negative ones. They reported practices such as prayer, Bible reading, positive mentalization, and efforts to connect with God and/or access spirituality.	Qualitative reports confirm the quantitative data of predominantly positive strategies.
High score on “Positive position before God” (4.58)	The thematic category “Believing in God strengthens me”: relief from suffering through faith in God” showed comments that characterize faith in God as the primary mechanism to face daily challenges of caring for a child who needs continuous and complex care.	Qualitative topics are consistent with the high and moderate scores.
Moderate scores for “Transformation of oneself and/or one’s life” (3.52) and “Offering to help another” (3.46)	The thematic category “Transformation in ways of thinking, feeling, and interacting with others and oneself” includes the incorporation of charitable practices through emotional and material support for those in need.	
Low scores on “Actions in search of spiritual help” (2.35) and “Search for support from Clergy” (3.19)	Family members reported distance from religious institutions due to care demands.	Quantitative results align with lower institutional engagement.
Higher negative SRCOPE score among younger family members (p = 0.014)	Feelings of revolt at child’s birth or diagnosis, especially among younger parents.	Reports of emotional distress at the time of diagnosis were consistent with the results of multiple linear regression model.
Higher positive SRCOPE score among family members living with fewer people (p = 0.006)	No qualitative data available to explain this result.	-

## DISCUSSION

In the present investigation, family members described a greater use of positive strategies of religious-spiritual coping versus negative ones. This finding is similar to that in a study conducted with informal caregivers of children with cleft lip and/or palate who were fed exclusively by gastrostomy tube, where positive SRCOPE reached a mean of 3.30, and negative SRCOPE, 1.88^(16)^. Moreover, it supports the qualitative reports of that study, which were mostly positive. In both studies, the importance attributed to religiosity and spirituality expressed in family members’ statements is reflected in high indexes of positive SRCOPE.

Another finding of this study was the statistically significant association between family member’s age and religious-spiritual coping. Younger family members obtained higher scores for negative SRCOPE. This finding was consistent with an integrative review, which found that advanced age influences religious-spiritual coping practice among informal caregivers, suggesting a greater appreciation of spirituality with maturation and processes experienced^(17)^. Another explanation for such an association may be the initial impact and denial of reality at the time of diagnosis, a phase in which parents are usually younger. For instance, one of the grandmothers participating in the qualitative stage who had experienced her grandson’s condition for eight years described an initial feeling of revolt at the time of the child’s birth, with a gradual strengthening and acceptance based on faith over the years.

In this study, the number of residents in the household ranged from two to six. The relationship between this variable and religious-spiritual coping was statistically significant, indicating a greater adoption of positive strategies among family members who lived with fewer people. In the literature, we found no study dealing with the effect of family size on religious-spiritual coping of caregivers of children with chronic conditions. However, this relationship has been observed in studies that analyzed overload and stress. According to an Iranian study conducted with 385 family members of children with chronic conditions, perceived burden was higher among families of four or more people^(18)^.

Among the positive strategies, “positive position before God” scored the highest, reaching a mean of 4.58. This factor refers to behaviors where an individual establishes a closer relationship with God for strength, support, and protection. The thematic category “Believing in God strengthens me”: relief from suffering through faith in God” supports this quantitative result, with comments that characterize faith in God as the primary mechanism to face the daily challenges of caring for a child who needs continuous and complex care. This finding is consistent with other studies of spirituality among caregivers. For instance, in a qualitative study conducted in Indonesia, parents and caregivers of children with special needs described faith or spirituality as a source of comfort, peace, and hope, believing that God is with them, which helped them have confidence to cope with the situation^(19)^. These caregivers also described religious rituals such as prayer and supplication to endure adversity, similar to the present study where family members listed prayer,

Bible reading, positive mentalization, and seeking to connect with God and/or access spirituality.

The statements that made up the thematic category “Gratitude to God and trust in divine purposes” indicated the belief in the existence of a higher force that is in control of all things and the hope that nothing is impossible before God, ratifying the literature on the subject. Religion and spirituality played a crucial role in the acceptance process of family members of children seen in a pediatric oncology outpatient clinic in Iran^(20)^. Belief in a God controlling life and disease represented an essential source of comfort to parents of children with rare lung disease in the United States of America^(21)^. Spirituality and belief in a higher being facilitated the adaptation of families facing a child’s multiple disabilities conditions, not only at the time of diagnosis but also during daily care^(22)^.

Family members in this study expressed a sense of hope for clinical improvement or definitive recovery of their children with continuous and complex care. Other researchers have analyzed narratives about hope among families of children with chronic diseases, describing this hope as of the expectant type, projecting a desired future, usually in search of “a normal and happy life”^(23)^. However, the authors note that hope is dynamic and is transformed throughout the experience of illness. Similarly, others authors found that parents of children with rare lung diseases initially sought divine intervention in search of an immediate miracle. Over time, they began to pray and give thanks for the small achievements of everyday life^(21)^.

Participants in this study described how caring for a child who needs continuous and complex care led them to reflect on their attitudes and seek a new purpose in life. In the statements of the category “Transformation in ways of thinking, feeling, and interacting with others and oneself”, they mentioned incorporating charitable practices through emotional and material support to those in needs. This finding also aligns with quantitative data, with high scores on the factors “transformation of oneself and/or one’s life” and “offering help to another”, with a mean of 3.52 and 3.46, respectively.

Another notable finding is the low scores for positive SRCOPE factors related to religious institutions, with a mean of 2.35 for “actions in search of spiritual help” and 3.19 for “search for support from Clergy”. The experiences described by family members in this study suggest that a child’s birth caused a certain distancing from religious institutions due to the exhausting demands of care required by a child.

According to the literature, specialized care, high level of surveillance, and caregiver burden result in social restriction of families of children who require continuous and complex care^(20,22)^. Moreover, many of these children are highly susceptible to infection; consequently, family members are restricted to the home to avoid exposure^(21)^. This social withdrawal may be a protective strategy for family members to avoid discriminatory behaviors and stigma toward children’s disabilities^(19)^. According to a qualitative meta-synthesis, social restraint may be an attempt by parents to protect themselves and their children from social acceptance standards set in society^(22)^.

It is also important to acknowledge the relevance of studies on religiosity and spirituality within Brazil’s sociocultural context , a country considered a rich mosaic woven from a diverse array of traditions and practices that reflect its complex history and cultural diversity^(24)^. In the present study, Evangelicals predominated, ratifying data from the latest Brazilian Institute of Geography and Statistics (In Portuguese, *Instituto Brasileiro de Geografia e Estatística* - IBGE) census^(25)^, which indicates that Brazil has become a predominantly Evangelical country. Nevertheless, Brazil remains characterized by a plurality of beliefs and religions. According to data from the 2022 IBGE census^(25)^, there are approximately 579,700 religious institutions in Brazil, reflecting the country’s significant religious diversity, which includes Catholic, Evangelical, Afro-Brazilian, Indigenous, Islamic, Jewish, and Buddhist institutions.

While the results of this study are consistent with existing literature, some limitations should be acknowledged. A limitation is the small sample size, combined with the use of a non-probabilistic convenience sampling method, which increases the risk of selection bias. Another limitation concerns the lack of integration between qualitative and quantitative data in the results section.

## CONCLUSION

Family members in this study used positive religious-spiritual coping strategies more frequently, especially those from smaller families. However, younger family members used negative religious-spiritual coping strategies more frequently. In line with these findings, caregivers revealed an experience marked by trust in God’s designs and relief from suffering through faith.

The findings of this study are consistent with existing literature on religiosity and spirituality among family members of children who require continuous and complex care. These results can inform direct intervention strategies by considering these families’ spiritual needs as an important element of their overall care needs. It is recommended that healthcare professionals, especially nurses, adopt a new paradigm of care that takes into account families’ spiritual and religious needs when planning their interventions.

## Data Availability

The entire dataset supporting the results of this study was published in the article itself.
