# Supplementary material for: Religious-spiritual coping among family members of children requiring continuous and complex care: a mixed-methods study
Source: Rev Esc Enferm USP. 2025 Nov 28;59:e20250143. doi: 10.1590/1980-220X-REEUSP-2025-0143en (PMC12670640; doi:10.1590/1980-220X-REEUSP-2025-0143en)
Supplement: Translation

# Translation: Coping religioso-espiritual entre familiares de niños que requieren cuidados continuos y complejos: un estudio de métodos mixtos*

**DOI:** 10.1590/1980-220X-REEUSP-2025-0143es

**Authors:** Bruna Josiane de Lima, Aline Cristiane Cavicchioli Okido, Wendy Sue Looman, Aline Helena Appoloni Eduardo, Fernanda Machado Silva-Rodrigues, Fabrine Aguilar Jardim, Regina Aparecida Garcia Lima

**Affiliations:** 1 Universidade Federal de São Carlos, Programa de Pós-Graduação em Enfermagem, São Carlos, SP, Brasil.; 2 Universidade de São Paulo, Escola de Enfermagem de Ribeirão Preto, Departamento de Enfermagem Materno-Infantil e Saúde Pública, Ribeirão Preto, SP, Brasil.; 3 University of Minnesota Twin Cities, School of Nursing, Minneapolis, United States.; 4 Universidade de São Paulo, Escola de Enfermagem, São Paulo, SP, Brasil.

**Keywords:** Niño, Familia, Religión, Espiritualidad, Enfermedad Crónica

## Abstract

**Objetivo:**

analizar el uso del *coping (afrontamiento)* religioso-espiritual entre los familiares de niños que requieren cuidados continuos y complejos.

**Métodos:**

se desarrolló un estudio de métodos mixtos en Brasil con familiares de niños que requieren cuidados continuos y complejos. En la etapa cuantitativa, 51 familiares respondieron a una encuesta demográfica y a la Escala Breve de Afrontamiento Espiritual/Religioso. En la etapa cualitativa, 15 familiares respondieron a una pregunta abierta sobre la práctica de la religiosidad y la espiritualidad en su vida cotidiana.

**Resultados:**

la edad de los familiares y el número de residentes en el hogar se asociaron significativamente con el coping religioso-espiritual. De las declaraciones surgieron tres categorías temáticas, que revelaron una experiencia marcada por la confianza en los designios de Dios y el alivio del sufrimiento que proporciona la fe.

**Conclusión:**

los familiares de este estudio utilizaron con mayor frecuencia estrategias de coping religioso-espiritual positivas, especialmente los de familias más pequeñas. Sin embargo, los familiares más jóvenes utilizaron con mayor frecuencia estrategias de coping religioso-espiritual negativas.

## INTRODUCCIÓN

Los niños que requieren cuidados continuos y complejos padecen afecciones médicas graves y limitaciones funcionales e intelectuales^(1,2)^. Este grupo incluye a niños con anomalías congénitas y enfermedades genéticas, prematuridad y complicaciones relacionadas, y parálisis cerebral^(3)^. Son comunes los dispositivos tecnológicos de soporte vital, como la gastrostomía y la traqueotomía^(2)^. Estos niños requieren una rutina de cuidados exhaustiva; en consecuencia, los padres y otros miembros de la familia experimentan cargas emocionales, físicas, sociales y económicas extremas^(4)^.

A pesar de estos retos, los familiares de los niños que requieren cuidados continuos y complejos buscan recursos para adaptarse a las circunstancias. La religiosidad y la espiritualidad constituyen elementos fundamentales de consuelo y fortaleza para superar las dificultades^(5)^. Estos términos están estrechamente relacionados, pero son conceptualmente distintos. La religiosidad abarca creencias y prácticas que a menudo se asocian con la adhesión a una tradición religiosa. La espiritualidad, por otro lado, es un concepto más amplio que se refiere a la relación humana con lo que se considera divino, sagrado o trascendente, y que implica una búsqueda de significado que no está necesariamente ligada a la religión institucional^(6)^.

El término «coping religioso-espiritual positivo» se refiere al uso de creencias, comportamientos o fe religiosos o espirituales para minimizar o tolerar el sufrimiento^(7)^. Por el contrario, el coping religioso-espiritual negativo se refiere al uso de estrategias que tienen repercusiones perjudiciales o negativas, como cuestionar la existencia de Dios, delegar la resolución del problema en Dios y entender una situación estresante como un castigo^(7)^.

Hay pruebas emergentes de que ciertas estrategias de coping religioso-espiritual se asocian con mejores resultados para los cuidadores. En un estudio realizado con cuidadores de niños con necesidades sanitarias especiales, los cuidadores familiares sin creencias religiosas tenían 2,7 veces más probabilidades de sufrir altos niveles de carga en comparación con los que profesaban una creencia religiosa^(8)^. Otras investigaciones revelaron que los cuidadores de niños con complejidad médica creían que la religión y la espiritualidad les ayudaban a afrontar la situación y a tomar decisiones informadas sobre la intervención médica de sus hijos^(9)^.

Según una revisión sistemática y un metaanálisis, las enfermeras han propuesto varias intervenciones en este ámbito en las últimas décadas. Se ha demostrado que estas intervenciones alivian el sufrimiento, aumentan la esperanza, ayudan a los pacientes a prepararse para las cuestiones relacionadas con el final de la vida, reducen los síntomas depresivos, mejoran la calidad de vida y el bienestar, disminuyen el estrés percibido y promueven la empatía. Los autores sostienen además que un enfoque de enfermería holístico debe incluir intervenciones espirituales, ya que el sufrimiento, el dolor y las experiencias negativas suelen ser inherentes a los problemas relacionados con la salud^(10)^.

Aunque el papel de la religiosidad y la espiritualidad en la atención médica ha recibido una atención creciente, sigue existiendo una brecha crítica en la comprensión de cómo los familiares de los niños que requieren cuidados continuos y complejos experimentan y utilizan estas dimensiones. Investigar el coping religioso-espiritual en este contexto es esencial para orientar la práctica de la enfermería y desarrollar estrategias de atención holística.

Para avanzar en la presente investigación, se propuso la siguiente pregunta de investigación: ¿cómo utilizan los familiares de los niños que requieren cuidados continuos y complejos el coping religioso-espiritual, teniendo en cuenta la influencia de las características sociodemográficas y sus experiencias cotidianas de religiosidad y espiritualidad?

En consecuencia, el objetivo de este estudio fue analizar el coping religioso-espiritual entre los familiares de los niños que necesitan cuidados continuos y complejos.

## MÉTODO

### DiseñO del estudio

Se trata de un estudio de método mixto de tipo explicativo secuencial^(11)^. Este enfoque se caracteriza por la recopilación y el análisis de datos cuantitativos en la primera etapa, seguidos de la recopilación y el análisis de datos cualitativos en la segunda etapa para complementar el conocimiento sobre el fenómeno estudiado. Se dio más peso a la etapa cuantitativa y menos a los datos cualitativos, para lo cual se utilizó la notación QUAN-qual (CUALITATIVO-cualitativo). La etapa cuantitativa se llevó a cabo como un estudio observacional, analítico y transversal, mientras que la etapa cualitativa siguió un diseño descriptivo y exploratorio.

Los resultados se integraron al final de la sección de resultados utilizando una matriz comparativa o de visualización conjunta, en la que se presentaron las categorías cualitativas junto con los resultados cuantitativos. Además, se utilizaron los conocimientos cualitativos en la sección de discusión para ayudar a interpretar los resultados cuantitativos.

### Población y criterios de selección

Los participantes eran familiares mayores de 18 años de niños de 0 a 12 años que requerían cuidados continuos y complejos y residían en una ciudad de la zona rural del estado de São Paulo, Brasil. En este estudio, se consideró familiar a cualquier pariente cercano que viviera con el niño y tuviera vínculos de afinidad y afecto (por ejemplo, abuelos, tíos). Para determinar si un niño requería cuidados continuos y complejos, se adoptó el siguiente criterio: el niño debía presentar al menos tres necesidades de cuidados: cuidados para el desarrollo, cuidados para la medicación, cuidados tecnológicos y/o cuidados rutinarios modificados^(12)^. El criterio de exclusión fue cualquier limitación intelectual que pudiera dificultar la cumplimentación de los instrumentos de recopilación de datos. No se realizó ningún cálculo del tamaño de la muestra para este estudio. Se utilizó una estrategia de muestreo no probabilístico por conveniencia.

### Recopilación De Datos

La recopilación de datos se llevó a cabo entre julio y noviembre de 2022. Las primeras participantes fueron reclutadas a partir de una lista de madres de niños que requerían cuidados continuos y complejos y que ya habían participado en un estudio anterior desarrollado por el grupo de investigación. Otra estrategia adoptada fue la difusión de la investigación en grupos de redes sociales como WhatsApp^p®^ y Facebook^®^. Cuando una posible participante expresaba su interés en participar en el estudio, se programaba una visita domiciliaria para la recopilación de datos. Durante la visita, también se invitó a participar a otros miembros de la familia que estaban presentes.

En la primera etapa (QUAN), los miembros de la familia completaron una encuesta desarrollada por los autores para recopilar los siguientes datos sociodemográficos: grado de parentesco con el niño; edad; estado civil; educación; ocupación; ingresos familiares; y número de residentes en el hogar. El cuidador principal (normalmente la madre) también respondió a preguntas sobre los niños que necesitan cuidados continuos y complejos, como la edad y el diagnóstico médico, con el fin de caracterizar más plenamente la muestra.

Posteriormente, los familiares respondieron a la Escala Breve de Afrontamiento (*Coping*) Espiritual/Religioso (Brief Scale for Spiritual/Religious Coping - SRCOPE), desarrollada inicialmente en Estados Unidos y traducida y validada al portugués brasileño^(7,13)^. Esta escala consta de 49 ítems subdivididos en 11 factores, siete relacionados con estrategias positivas y cuatro con estrategias negativas. Esta medida permite calcular cuatro índices: SRCOPE positivo: media de 34 ítems relacionados con la dimensión positiva; SRCOPE negativo: media de 15 ítems relacionados con la dimensión negativa; ratio SRCOPE negativo/SRCOPE positivo: porcentaje obtenido al calcular la ratio entre el SRCOPE negativo y el SRCOPE positivo; y SRCOPE total: media de la suma entre el SRCOPE positivo invertido y el SRCOPE negativo.

Los valores resultantes oscilan entre 1 y 5, donde los valores más altos reflejan un mayor uso de la estrategia (SRCOPE positivo, negativo y total), excepto en el caso del índice de relación, que oscila entre 0,2 y 5, donde los valores más bajos indican un mayor uso del SRCOPE positivo en contraposición al SRCOPE negativo. El análisis factorial, de consistencia interna y de correlación indicó que la Escala SRCOPE brasileña es un instrumento válido y confiable. La versión brasileña mostró un excelente nivel de consistencia interna, y el alfa de Cronbach fue de 0.98 para la dimensión SRCOPE positiva y de 0.86 para la dimensión SRCOPE negativa^(13)^.

En la segunda etapa, se aplicaron los mismos criterios de inclusión y se invitó a todos los familiares elegibles a responder a la siguiente pregunta abierta: ¿cómo están presentes la espiritualidad y la religión en su vida cotidiana? De los 51 familiares que participaron en la primera etapa, 15 estuvieron dispuestos a informar sobre su experiencia en la segunda etapa. El tiempo aproximado de recopilación fue de 20 minutos por participante.

### Análisis Y Tratamiento De Datos

Para el análisis de la etapa QUAN, se consideraron variables dependientes los cuatro índices que podían calcularse mediante el SRCOPE, tal y como se ha descrito anteriormente. Las variables independientes fueron las obtenidas de la aplicación del instrumento sociodemográfico, que se subdividió en variables categóricas (grado de parentesco, religión, educación, ocupación y estado civil) y variables continuas (edad de los familiares, ingresos familiares y número de residentes en el hogar). Los análisis estadísticos se realizaron en el Sistema de Análisis Estadístico versión 9.2 para Windows. Se utilizó un análisis de regresión lineal simple y múltiple con criterios de selección por pasos^(14)^. Las diferencias entre las variables se consideraron estadísticamente significativas cuando p < 0,05.

En la segunda etapa del análisis, las entrevistas se transcribieron y examinaron utilizando un método de análisis de contenido inductivo^(15)^, siguiendo las fases de preanálisis, exploración del material, tratamiento de los resultados, inferencia e interpretación. Dos investigadores realizaron la codificación de los datos de forma manual y se consultó a un tercer investigador para resolver cualquier discrepancia antes del proceso final de lectura y codificación. Del análisis surgieron tres categorías temáticas: i) «Creer en Dios me fortalece»: alivio del sufrimiento a través de la fe en Dios; ii) Transformación en las formas de pensar, sentir e interactuar con los demás y con uno mismo; iii) Gratitud a Dios y confianza en los designios divinos.

El Cuadro 1 resume cómo se desarrollaron las categorías temáticas a partir de los comentarios de los participantes, agrupados mediante códigos iniciales e intermedios.

**Cuadro 1:** Resumen del proceso analítico de los datos cualitativos – São Carlos, SP, Brasil, 2022

Códigos iniciales	Códigos intermedios	Categorías temáticas
*Creer en Dios me fortalece.* *Renueva nuestras fuerzas cada día.* *Dios es nuestro refugio y nuestra fortaleza, siempre.*	La fe ayuda a afrontar los problemas cotidianos	
*Dios lo es todo en nuestras vidas.* *Sin Dios y Jesucristo, no soy nada. Sin Él, hoy no estaría aquí.*	Dios en el centro	
*Mentalización* *Clamor sincero.* *Oración.* *Oraciones y súplicas.*	Estrategia para conectar con Dios y acceder a la espiritualidad	«Creer en Dios me fortalece»: alivio del sufrimiento a través de la fe en Dios
*Hay momentos en los que pensamos que estamos solos y que no vamos a tener* éxito *Lloro como cualquier ser humano.* *Hay días en los que estamos deprimidos.* Me debilité. *Me enfadé.*	Momentos de debilidad	
*El niño vino a mejorarme como persona.* Nunca sería la persona que soy hoy si Dios no me los hubiera dado. Intento controlarme para ser mejor	Reflexión sobre sus actitudes	
*Amar al prójimo, ejercer la caridad.* *Dentro de una ONG que ayuda a las madres* *Ayudar a las personas con apoyo psicológico, prestándoles atención y cuidados*	Ofrecer ayuda a los demás	Transformación en la forma de pensar, sentir e interactuar con los demás y con uno mismo	
*No soy una persona religiosa; creé mi iglesia dentro de mi casa.* *No me aferro a ninguna religión; me aferro a Dios.* *Dios, Jesús está por encima de cualquier religión.* *Dejé de ir a la iglesia, pero tengo mucha fe en Dios.* *No soy una persona 100 % religiosa.*	Dando un nuevo significado a la religión y/o a las instituciones religiosas	
*Estoy agradecido de que Dios haya confiado en mí para cuidarles, amarles y protegerles.* *Todo tiene un propósito.* *Confío en* Él	Gratitud a Dios.	
*Si Dios quiere llevárselos, lo aceptaré.* *Si ya habían cumplido su misión, podían irse.* *Ya no necesitaban sufrir.* *Esto algún día llegará a su fin.*	Aceptación de la terminalidad	Gratitud a Dios y confianza en los propósitos divinos
*Creer en los milagros.* *Serán niños totalmente normales.* *Para Dios, nada es imposible.*	*Esperanza de mejora*

### Aspectos Éticos

Este estudio cumplió con las normas relativas a la investigación con seres humanos establecidas en la Resolución 510/16 del Consejo Nacional de Salud de Brasil. El proyecto fue aprobado por el Comité de Ética en Investigación (Proceso 5.507.466, de fecha 4 de julio de 2022). Para preservar el anonimato, los participantes en la fase cualitativa fueron identificados mediante una codificación alfanumérica, basada en el orden cronológico de su participación, de la siguiente manera: P1, P2, y así sucesivamente. Los participantes que aceptaron participar en el estudio fueron informados y firmaron el formulario de consentimiento informado.

## RESULTADOS

### Primera etapa(QUAN)

En la primera etapa cuantitativa (QUAN) participaron 51 familiares de 28 niños que requerían cuidados continuos y complejos. Entre los participantes se encontraban 28 madres, ocho tíos, siete abuelos, cinco padres, dos padrastros y una hermana. La edad media de los familiares era de 40,3 años, con un rango de 18 a 77 años. La mayoría de los participantes (80,4 %) declararon tener pareja. En cuanto a la ocupación y la educación, 32 (62,8%) tenían empleo y 27 (52,9%) habían completado la educación secundaria. La renta familiar media era de 3680,00 R$ (*reais*, moneda de Brasil). El ingreso familiar promedio *per cápita* fue de 1001,00 reales, con un rango de 166,00 a 4000,00 reales por persona. En promedio, los hogares tenían 3,7 residentes, con un mínimo de dos y un máximo de seis. En cuanto a la religión, los cristianos evangélicos fueron el grupo más representado (48,8%), seguidos por los católicos.

En cuanto a los niños que requerían cuidados continuos y complejos, la edad media era de 5,8 años, dividida por igual

entre ambos sexos. En cuanto al diagnóstico, 14 (50%) niños tenían trastornos neurológicos; nueve (32%) tenían secuelas graves debido a la prematuridad; y cinco (18%) tenían síndromes genéticos y/o anomalías congénitas.

La Tabla 1 presenta la media, la desviación estándar, el mínimo, el máximo, la mediana y los valores cuartiles de los cuatro índices y los 11 factores calculados por el SRCOPE. Este análisis identificó que los familiares utilizaban más estrategias positivas que negativas, con una media de la relación SRCOPE negativo/SRCOPE positivo que se mantenía en 0,56. Los familiares utilizaron con mayor frecuencia el factor positivo «posición positiva ante Dios», alcanzando una media de 4,58. Sin embargo, el factor positivo «acciones en busca de ayuda espiritual» tuvo la media más baja entre los familiares de este estudio (2,35).

**Tabla 1:** Coping religioso-espiritual de los familiares de niños que necesitan cuidados continuos y complejos (n = 51) según la media, la desviación estándar, el valor mínimo, el valor máximo, la mediana y los cuartiles – São Carlos, SP, Brasil, 2022.

Variables	Media	DE	Mín.	C1	Mediana	C3	Máx.
Índices	
SRCOPE positivo	3,28	0,51	2,03	3,03	3,32	3,65	4,00
SRCOPE negativo	1,82	0,63	1,00	1,40	1,73	2,07	3,93
Total SRCOPE	3,73	0,40	2,75	3,51	3,83	3,96	4,49
Relación SRCOPE negativo/SRCOPE positivo	0,56	0,22	0,25	0,43	0,50	0,61	1,23
Factores positivos	
Factor P1: Transformación de uno mismo y/o de la propia vida	3,52	0,79	1,77	3,16	3,67	3,94	4,88
Factor P2: Acciones en busca de ayuda espiritual	2,35	0,98	1,0	1,8	2,4	2,8	4,8
Factor P3: Ofrecerse a ayudar a otra persona	3,46	0,93	1,4	3	3,4	4,2	5,0
Factor P4: Posición positiva ante Dios	4,58	0,61	1,8	4,3	4,8	5,0	5
Factor P5: Búsqueda de apoyo por parte del clero	3,19	0,89	1,0	2,75	3,25	4,0	4,75
Factor P6: Entrega activa a través de Dios/la religión/la espiritualidad	4,08	0,90	1,67	3,67	4,33	5,0	5	
Factor P7: Búsqueda del conocimiento espiritual	2,98	0,93	1,0	2,33	3,0	3,66	5,0
Factores negativos	
Factor N1: reevaluación negativa de Dios	1,52	0,85	1	1	1	1,8	5,0
Factor N2: Posición negativa ante Dios	2,55	0,99	1,0	2,0	2,33	3,0	5
Factor N3: Insatisfacción con el clero	1,47	0,95	1,0	1	1	1,37	4,75
Factor N4: reevaluación negativa del significado	2,00	1,05	1	1	1,67	2,66	4,67

Leyenda: DE: desviación estándar; Mín.: valor mínimo; Máx.: valor máximo. C1 (1er cuartil) C3 (3er Cuartil)

La Tabla 2 muestra el efecto de las variables sociodemográficas en el coping religioso-espiritual de los familiares de niños que requieren cuidados continuos y complejos según el modelo de regresión lineal simple. Se encontró una asociación negativa estadísticamente significativa entre el SRCOPE negativo y variables como la edad de los familiares y los ingresos familiares. Cuanto mayor era la edad y los ingresos, menor era la adopción de estrategias negativas. Se identificó otra asociación negativa entre el número de residentes en el hogar, el SRCOPE positivo y los índices totales de SRCOPE, lo que indica que las personas de hogares con menos miembros obtuvieron puntuaciones más altas en estrategias positivas. No se identificaron relaciones significativas entre las variables categóricas.

**Tabla 2:** Efecto de las variables sociodemográficas en el coping religioso-espiritual de los familiares de niños que necesitan cuidados continuos y complejos (n = 51), según el modelo de regresión lineal simple – São Carlos, SP, Brasil, 2022.

		SRCOPE positivo			SRCOPE negativo			SRCOPE total			Relación SRCOPE negativo/SRCOPE positivo		
Variables	Categorías	Beta (EE)	p	R^2^	Beta (EE)	p	R^1^	Beta (EE)	p	R^2^	Beta (EE)	p	R^2^
Parentesco	Madre (ref.) Otros miembros de la familia	– -5.27 (4.16)	0.211	0.0317	– -7.17 (4.09)	0.086	0.0589	– 2.38 (4.21)	0.575	0.0065	– -4.04 (4.19)	0.339	0.0186
Edad de la familia	Variable continua	0.06 (0.14)	0.674	0.0036	-0.34 (0.13)	**0.014**	0.1177	0.33 (0.14)	**0.019**	0.1065	-0.33 (0.13)	**0.018**	0.1095
Religión	Católico (ref.) Evangélico Otras	– -4.53 (6.11) -6.46 (6.71)	0.463 0.342	0.0246	– 4.86 (6.14) 3.44 (6.74)	0.433 0.612	0.0163	– -6.21 (5.86) -9.19 (6.44)	0.296 0.162	0.0520	– 6.79 (6.00) 7.46 (6.59)	0.265 0.265	0.0398
Educación	Escuela primaria (ref.) Secundaria Educación superior	– -6.66 (7.28) -2.35 (7.51)	0.365 0.756	0.0285	– 2.67 (7.23) -3.53 (7.47)	0.713 0.638	0.0390	– -5.81 (7.23) 0.21 (7.47)	0.425 0.978	0.0412	– 5.46 (7.20) -1.31 (7.44)	0.453 0.861	0.0491
Estado civil	Con pareja (ref.) Sin pareja	– -1.87 (5.29)	0.726	0.0025	– -1.68 (5.28)	0.752	0.0021	– 1.18 (5.29)	0.824	0.0010	– -1.62 (5.29)	0.761	0.0019
Ocupación	Autónomo (ref.) Contrato firmado Hogar/asalariado Desempleado	– -6.45 (5.55) 0.51 (5.84) 0.71 (9.72)	0.251 0.930 0.942	0.0520	– -0.72 (5.49) -1.44 (5.78) 15.85 (9.61)	0.896 0.804 0.106	0.0722	– -3.51 (5.46) 2.95 (5.75) -14.36 (9.56)	0.524 0.610 0.140	0.0834	– 4.16 (5.33) -3.40 (5.61) 18.39 (9.33)	0.440 0.548 0.055	0.1276
Ingresos familiares	Variable continua	-0.08 (0.14)	0.573	0.0065	-0.29 (0.14)	**0.037**	0.0860	0.19 (0.14)	0.182	0.0361	-0.24 (0.14)	0.094	0.0563
Residentes en el hogar	Variable continua	-0.44 (0.15)	**0.006**	0.1581	0.30 (0.15)	0.056	0.0788	-0.51 (0.15)	**0.001**	0.2088	0.42 (0.15)	**0.008**	0.1460

Leyenda: beta: valor de la estimación o coeficiente angular (pendiente) en la línea de regresión; EE: error estándar de beta; R^2^: coeficiente de determinación (% de la variabilidad de la variable de respuesta explicada por la variable independiente). Las variables sin distribución normal se transformaron en rangos.

Finalmente, las variables estadísticamente significativas se introdujeron en un modelo de regresión lineal múltiple mediante regresión logística por pasos hacia atrás (Wald), como se muestra en la Tabla 3. Las relaciones entre la «edad de la familia» y el SRCOPE negativo, y entre los «residentes en el hogar» y el SRCOPE positivo, el SRCOPE total y la relación SRCOPE negativo/SRCOPE positivo se mantuvieron en el modelo con significación estadística. En resumen, los miembros más jóvenes de la familia obtuvieron puntuaciones más altas en SRCOPE negativo, y aquellos que dijeron que vivían con menos personas tenían mejores índices de estrategias de coping religioso-espiritual positivo.

**Tabla 3:** Efecto de las variables sociodemográficas en el coping religioso-espiritual de los familiares de niños que necesitan cuidados continuos y complejos (n = 51) según el modelo de regresión lineal múltiple – São Carlos, SP, Brasil, 2022

Variables		Beta (EE)	p	R parcial
SRCOPE positivo	Residentes en el hogar	-0,44 (0,15)	**0,006**	0,1581
SRCOPE negativo	Edad de la familia	-0,34 (0,13)	**0,014**	0,1177
SRCOPE total	Residentes en el hogar	-0,51 (0,15)	**0,001**	0,2088
Relación SRCOPE negativo/SRCOPE positivo	Residentes en el hogar	0,42 (0,15)	**0,008**	0,1460

Leyenda: beta: valor de la estimación o coeficiente angular (pendiente) en la línea de regresión; EE: error estándar de beta; R^2^: coeficiente de determinación, criterio escalonado de selección de variables; R^2^parcial: 0,1581; intersección (SE): 37,05 (4,10); P < 0,001. Las variables sin distribución normal se transformaron en rangos.

### Segunda Etapa (Qual)

En la segunda etapa -cualitativa- participaron un total de 11 madres, una abuela, una tía, un padre y un tío, lo que suma un total de 15 familiares de 13 niños que necesitan cuidados continuos y complejos. Sus testimonios permitieron construir tres categorías temáticas, que se presentan a continuación.

«*Creer en Dios me fortalece»: alivio del sufrimiento a través de la fe en Dios*

Al relatar sus experiencias, las familias cuyos comentarios se incluyeron en la primera categoría temática hicieron hincapié en la creencia en una fuerza superior traducida como «fe» que les ayudó a afrontar «momentos difíciles», como el impacto del diagnóstico.

*En los momentos difíciles, por ejemplo, el diagnóstico, que fue un momento que me conmocionó mucho, busqué la fe, busqué la fuerza en Dios* [...] *porque creer en Dios me fortalece, me da confianza, me hace creer que, a pesar de todo,* Él *está conmigo.* (P8, madre de un niño de 10 años con trastorno grave del espectro autista)

*Cuando enfrento momentos difíciles en la vida, me sorprendo rezando; digo mis oraciones y les pido que me fortalezcan y me ayuden a enfrentar esa situación.* (P14, madre de un niño de 6 años con parálisis cerebral)

El momento de descubrir la condición de un niño suele estar impregnado de sentimientos negativos hacia uno mismo, como culpa, rebeldía, impotencia y dolor, que son sentimientos clasificados como coping religioso-espiritual negativo. Sin embargo, incluso en momentos de gran fragilidad, estos familiares describieron la importancia de la «fe en Dios» para obtener fuerzas y seguir adelante.

*Desde el momento en que nació, sentí que algo no iba bien, pero no podía explicar qué estaba pasando. Me sentía muy* débil; *pensaba que todo había sido causado por un «culpable*», que todo *estaba mal, y cuando lo veía, algo me rebelaba ,no tenía fuerzas. Hoy digo que me faltaba fe.* (P1, abuela de un niño de 8 años con parálisis cerebral)

*Hay momentos en los que incluso pensamos que estamos solos y que no lo conseguiremos. A veces, incluso lloro como cualquier ser humano, pero luego vuelvo a mentalizarme, a fortalecerme y a creer en ese ser superior, nuestro Dios.* (P14, madre de un niño de 6 años con parálisis cerebral)

Dado que las exigencias que se imponen a los niños que requieren cuidados continuos y complejos son un reto permanente, la fe también se describió como una base para afrontar la rutina diaria.

*Todo depende de nuestra fe para ayudarnos a calmarnos, porque el día a día es un reto con estos niños.* (P3, madre de un niño de 9 años con trastorno grave del espectro autista)

*Es la fe la que nos da esperanza para el mañana* [...] *la fe es un pilar; es la base, es el fundamento de mi vida; sin ella, no podría afrontar los problemas cotidianos.* (P7, tía de un niño de 5 años con trastorno grave del espectro autista)

*Dios está siempre presente en mi vida cotidiana; tengo mucha fe y siempre pienso en positivo. Hay días en los que estamos deprimidos, ya sabes, somos seres humanos, pero al día siguiente me levanto y creo que Dios me ha dado una nueva oportunidad para respirar, así que cuando estamos agradecidos por las pequeñas cosas, Dios está presente en todo.* (P5, madre de un niño de 6 años con trastorno grave del espectro autista)

*Mife en el Señor Jesús es lo que me mantiene en pie. Todo está en manos del Señor. La fe nos lleva mucho* más *allá de lo que podemos imaginar. Creo que renueva nuestras fuerzas cada día.* (P15, madre de un niño de 7 años con parálisis cerebral)

*Creyendo en Dios, con fe, me despierto cada día. Dios lo es todo en nuestras vidas.* (P11, abuelo de un niño de 4 años con parálisis cerebral)

A continuación, se presentan ejemplos de estrategias para conectarse con Dios y/o acceder a la espiritualidad descritas por los familiares:

*Ya me despierto por la mañana y rezo, dando gracias a Dios por la noche y por mi salud.* (P4, madre de un niño de 3 años con trastorno grave del espectro autista)

*Todos los días leo la Biblia, me detengo a rezar e intento conectarme. Intento seguir esta* línea [...] por supuesto *que fallamos, soy humano, pero este es mi propósito, mi dirección.* (P9, padre de un niño de 10 años con trastorno grave del espectro autista)

*Siempre que lo necesito,* Él *está en mi vida. Sí, solo clamo desde mi corazón y* Él *siempre me ayuda.* (P13, madre de un niño de 7 años con parálisis cerebral)

*Transformación en la forma de pensar, sentir e interactuar con los demás y con uno mismo*

Algunos familiares interpretaron la experiencia de cuidar a un niño que necesita cuidados continuos y complejos como una posibilidad de crecimiento espiritual y transformación personal.

*Creo que nunca sería la persona que soy hoy si Dios no me lo hubiera dado.* (P1, abuela de un niño de 8 años con parálisis cerebral)

*Mi hija es mi milagro divino. Ha renacido de nuevo y yo he aprendido mucho de los retos a los que me enfrento a diario.*(P12, madre de un niño de 5 años con síndrome de Guillain-Barré)

Los familiares también compartieron ejemplos de transformaciones en su forma de actuar a través de prácticas caritativas, ya sea mediante oraciones, apoyo emocional o incluso apoyo material a los necesitados.

*Veo que todo tiene un propósito. Si no hubiera tenido que pasar por todo lo que estoy pasando, nunca estaría dentro de una ONG ayudando a otras madres.* (P1, abuela de un niño de 8 años con parálisis cerebral)

*Intento aplicar el Evangelio de Jesús en mi vida, amando a mi prójimo, ejerciendo la caridad, siendo mejor hoy que ayer e intentando controlarme para ser mejor.* (P6, madre de un niño de 10 años con parálisis cerebral)

*Dentro de la religión y la espiritualidad, intento ayudar a las personas y escuchar a quienes lo necesitan; así es como sigo adelante y obtengo la fuerza para seguir adelante.* (P14, madre de un niño de 6 años con parálisis cerebral)

*Rezo mis oraciones y devociones; pido por mí y por mi familia, amigos y vecinos. Creo que las madres deben unirse y ayudarse mutuamente para ayudar a sus hijos. Si encuentra a alguna madre que quiera hablar, puede darle mi número.*(P3, madre de un niño de 9 años con trastorno grave del espectro autista)

La agotadora demanda de cuidados que requieren estos niños y las dificultades relacionadas con la movilidad de un lugar a otro afectaron a la relación de los miembros de la familia con las instituciones religiosas.

*Creo mucho en la Iglesia católica, pero con* él*, en particular, creé mi iglesia dentro de mi casa. Mi opinión es que Dios está en todas partes.* (P1, abuela de un niño de 8 años con parálisis cerebral)

Siempre fui estudiante de la Biblia de los Testigos de Jehová, pero después de que naciera mi hija, dejé de estudiar, dejé de ir a las iglesias. Dejé todo, pero tengo mucha fe en Dios, aunque no vaya a la iglesia, aunque no asista a la iglesia, le pido a Dios en lo más profundo de mi ser, y eso es todo. (P13, madre de un niño de 7 años con parálisis cerebral)

Los siguientes testimonios demuestran cómo algunos familiares valoran más la dimensión «espiritual» que la dimensión «religiosa»:

*La gente se aferra a Dios; yo no me aferro a ninguna religión, me aferro a Dios.* (P2, madre de un bebé de 8 meses con parálisis cerebral)

*Para mí, Dios, Jesús, está por encima de cualquier religión o iglesia. Cuando tienes fe y* Él *está en tu corazón,* Él *caminará contigo dondequiera que estés.* (P5, madre de un niño de 6 años con trastorno grave del espectro autista)

*No soy 100 % religiosa, pero tengo mucha fe y creo en un ser supremo, que es Dios. Creo en María, nuestra madre, y también creo mucho en la espiritualidad.* (P14, madre de un niño de 6 años con parálisis cerebral)

*Gratitud a Dios y confianza en los designios divinos*

La fe en Dios y la confianza en los designios divinos han ayudado a los miembros de la familia a desarrollar sus recursos para afrontar la experiencia de tener un hijo que necesita cuidados continuos y complejos.

*Así que, desde que era pequeña, siempre creí que había un Dios que me amaba y cuidaba de mí y que cualquier situación que ocurriera en mi vida, fuera buena o mala, era porque* Él *lo permitía y estaría a mi lado.* (P8, madre de un niño de 10 años con un trastorno grave del espectro autista)

Él *sabe que me pongo en sus manos y que se haga su voluntad. Esto me ayuda, me da un poco de fuerza, porque también* sé que *puedo contar con Dios.* (P13, madre de un niño de 7 años con parálisis cerebral)

Algunos familiares explicaron cómo la creencia de que nada es imposible para Dios fortalece sus sentimientos de esperanza con respecto a la mejoría clínica o la recuperación definitiva de un niño.

*Si Dios quiere, pronto nos darán el alta, pronto nos quitará todo esto* [refiriéndose a los dispositivos como el catéter y el oxígeno, por ejemplo]. *Pronto será un niño totalmente normal, si Dios quiere, y espero que esto se acerque mucho a lo que imagino, eso es todo lo que espero*. (P2, madre de un bebé de 8 meses con parálisis cerebral)

*Siempre luchó por la vida, así que creo que nuestra fe y su fuerza para sobrevivir, su voluntad de sobrevivir, nos hacen superar todos los retos. Nuestra fe nos hace creer en los milagros, en que en algún momento ella podrá superarlo y mostrar una mejora significativa y evolucionar mucho*. (P10, madre de un niño de 4 años con parálisis cerebral)

Además, incluso cuando se enfrentan a las adversidades y los retos que impregnan la vida cotidiana de estas familias y niños, expresan su gratitud a Dios por sus logros.

*Cada día, cuando me despierto y veo que* él *está despierto, me siento agradecida; mi día ya ha valido la pena. No importa si no hay nada para comer o no, lo que pase durante el día, siempre y cuando* él *se despierte.* (P1, abuela de un niño de 8 años con parálisis cerebral)

*Hoy solo tengo cosas por las que estar agradecida. Está ganando peso, interactúa y le va bien. Creo que lo único que tengo que hacer durante el resto de mi vida es estar agradecida por* él*.* (P2, madre de un bebé de 8 meses con parálisis cerebral)

*Estoy agradecida por todo lo que estoy logrando en beneficio de mi hijo. Creo que Dios me está ayudando. Dios me está guiando.* (P3, madre de un niño de 9 años con trastorno grave del espectro autista)

*Siempre en mis oraciones, le doy gracias a Dios por haberme concedido el honor de ser madre, porque tuve tres abortos espontáneos y me sometí a muchos tratamientos para poder tener a mi hijo.* (P6, madre de un niño de 10 años con parálisis cerebral)

Por último, algunos familiares describieron cómo la aceptación de la terminalidad también está impregnada de la creencia en los designios de Dios. Como señalaron estos participantes, esto está motivado principalmente por el deseo de poner fin al sufrimiento físico que suelen experimentar los niños que necesitan cuidados continuos y complejos.

*A veces la gente dice que soy fría cuando digo que si Dios quiere llevárselo, lo aceptaré porque no acepto el sufrimiento; no quiero verlo sufrir. Si tengo que verlo sufrir, acepto su partida. Llegará el dolor de la pérdida, pero el sufrimiento es peor.* (P1, abuela de un niño de 8 años con parálisis cerebral)

*También le he preguntado a Dios si ya había cumplido su misión; que podía irse para no tener que sufrir* más. (P2, madre de un bebé de 8 meses con parálisis cerebral)

Por último, el Cuadro 2 siguiente presenta una matriz comparativa, en la que se muestran los resultados cualitativos junto con los resultados cuantitativos.

**Table:** 

Resultados cuantitativos	Resultados cualitativos	Interpretación
Media de SRCOPE positivo: 3,28 Media de SRCOPE negativo: 1,82 Ratio: 0,56	Los familiares describieron un mayor uso de estrategias positivas de coping religioso-espiritual frente a las negativas. Los informes cualitativos confirman Informaron de prácticas como la oración, la lectura de la Biblia, la mentalización positiva y los esfuerzos por conectar predominantemente positivas con Dios y/o acceder a la espiritualidad.	Los informes cualitativos confirman los datos cuantitativos de estrategias predominantemente positivas.	
Puntuación alta en «Posición positiva ante Dios» (4,58)	La categoría temática «Creer en Dios me fortalece: alivio del sufrimiento a través de la fe en Dios» mostró comentarios que caracterizan la fe en Dios como el mecanismo principa para afrontar los retos diarios del cuidado de un niño que necesita cuidados continuos y complejos.	Los temas cualitativos son coherentes con las puntuaciones altas y moderadas.
Puntuaciones moderadas para «Transformación de uno mismo y/o de la propia vida» (3,52) y «Ofrecer ayuda a los demás» (3,46)	La categoría temática «Transformación en la forma de altas y moderadas. pensar, sentir e interactuar con los demás y con uno mismo» incluye la incorporación de prácticas caritativas a través del apoyo emocional y material a los necesitados.
Puntuaciones bajas en «Acciones en busca de ayuda espiritual» (2,35) y «Búsqueda de apoyo del clero» (3,19).	Los familiares informaron de un distanciamiento de las instituciones religiosas debido a las exigencias del cuidado.	Los resultados cuantitativos concuerdan con un menor compromiso institucional.
Puntuación SRCOPE negativa más alta entre los miembros más jóvenes de la familia (p = 0,014).	Sentimientos de rebelión ante el nacimiento o el diagnóstico Los informes de angustia emocional del niño, especialmente entre los padres más jóvenes.	Los informes de angustia emocional del niño, en el momento del diagnóstico coincidieron con los resultados del modelo de regresión lineal múltiple.
Puntuación SRCOPE positiva más alta entre los familiares que vivían con menos personas (p = 0,006).	No hay datos cualitativos disponibles para explicar este resultado.

## DISCUSIÓN

En la presente investigación, los familiares describieron un mayor uso de estrategias positivas de coping religioso-espiritual frente a las negativas. Este hallazgo es similar al de un estudio realizado con cuidadores informales de niños con labio leporino y/o paladar hendido que se alimentaban exclusivamente por sonda gástrica, en el que el SRCOPE positivo alcanzó una media de 3,30 y el SRCOPE negativo, 1,88^(16)^. Además, respalda los informes cualitativos de ese estudio, que fueron en su mayoría positivos. En ambos estudios, la importancia atribuida a la religiosidad y la espiritualidad expresada en las declaraciones de los familiares se refleja en los altos índices de SRCOPE positivo.

Otro hallazgo de este estudio fue la asociación estadísticamente significativa entre la edad de los familiares y el coping religioso-espiritual. Los familiares más jóvenes obtuvieron puntuaciones más altas en SRCOPE negativo. Este hallazgo fue coherente con una revisión integradora, que encontró que la edad avanzada influye en la práctica del coping religioso-espiritual entre los cuidadores informales, lo que sugiere una mayor apreciación de la espiritualidad con la maduración y los procesos experimentados^(17)^. Otra explicación de esta asociación puede ser el impacto inicial y la negación de la realidad en el momento del diagnóstico, una fase en la que los padres suelen ser más jóvenes. Por ejemplo, una de las abuelas que participó en la etapa cualitativa y que había vivido la enfermedad de su nieto durante ocho años describió un sentimiento inicial de rebeldía en el momento del nacimiento del niño, que se fue fortaleciendo y aceptando gradualmente a lo largo de los años gracias a la fe.

En este estudio, el número de residentes en el hogar oscilaba entre dos y seis. La relación entre esta variable y el coping religioso-espiritual fue estadísticamente significativa, lo que indica una mayor adopción de estrategias positivas entre los miembros de la familia que vivían con menos personas. En la literatura, no encontramos ningún estudio que tratara el efecto del tamaño de la familia en el coping religioso-espiritual de los cuidadores de niños con enfermedades crónicas. Sin embargo, esta relación se ha observado en estudios que analizaron la sobrecarga y el estrés. Según un estudio iraní realizado con 385 familiares de niños con enfermedades crónicas, la carga percibida era mayor entre las familias de cuatro o más personas^(18)^.

Entre las estrategias positivas, la «posición positiva ante Dios» obtuvo la puntuación más alta, alcanzando una media de 4,58. Este factor se refiere a los comportamientos en los que un individuo establece una relación más estrecha con Dios para obtener fuerza, apoyo y protección. La categoría temática «Creer en Dios me fortalece: alivio del sufrimiento a través de la fe en Dios» respalda este resultado cuantitativo, con comentarios que caracterizan la fe en Dios como el mecanismo principal para afrontar los retos diarios del cuidado de un niño que necesita cuidados continuos y complejos. Este hallazgo es coherente con otros estudios sobre la espiritualidad entre los cuidadores. Por ejemplo, en un estudio cualitativo realizado en Indonesia, los padres y cuidadores de niños con necesidades especiales describieron la fe o la espiritualidad como una fuente de consuelo, paz y esperanza, creyendo que Dios está con ellos, lo que les ayudó a tener confianza para afrontar la situación^(19)^. Estos cuidadores también describieron rituales religiosos como la oración y la súplica para soportar la adversidad, de manera similar al presente estudio, en el que los familiares mencionaron la oración, la lectura de la Biblia, la mentalización positiva y la búsqueda de la conexión con Dios y/o el acceso a la espiritualidad.

Las declaraciones que conformaban la categoría temática «Gratitud a Dios y confianza en los designios divinos» indicaban la creencia en la existencia de una fuerza superior que controla todas las cosas y la esperanza de que nada es imposible ante Dios, lo que ratifica la literatura sobre el tema. La religión y la espiritualidad desempeñaron un papel crucial en el proceso de aceptación de los familiares de los niños atendidos en una clínica ambulatoria de oncología pediátrica en Irán^(20)^. La creencia en un Dios que controla la vida y la enfermedad representaba una fuente esencial de consuelo para los padres de niños con enfermedades pulmonares raras en los Estados Unidos de América^(21)^. La espiritualidad y la creencia en un ser superior facilitaron la adaptación de las familias que se enfrentaban a las múltiples discapacidades de sus hijos, no solo en el momento del diagnóstico, sino también durante los cuidados diarios^(22)^.

Los familiares de este estudio expresaron una sensación de esperanza en la mejora clínica o la recuperación definitiva de sus hijos con cuidados continuos y complejos. Otros investigadores han analizado narrativas sobre la esperanza entre las familias de niños con enfermedades crónicas, describiendo esta esperanza como del tipo expectante, que proyecta un futuro deseado, normalmente en busca de «una vida normal y feliz»^(23)^. Sin embargo, los autores señalan que la esperanza es dinámica y se transforma a lo largo de la experiencia de la enfermedad. De manera similar, otros autores descubrieron que los padres de niños con enfermedades pulmonares raras buscaban inicialmente la intervención divina en busca de un milagro inmediato. Con el tiempo, comenzaron a rezar y a dar gracias por los pequeños logros de la vida cotidiana^(21)^.

Los participantes en este estudio describieron cómo el cuidado de un niño que necesita cuidados continuos y complejos les llevó a reflexionar sobre sus actitudes y a buscar un nuevo propósito en la vida. En las declaraciones de la categoría «Transformación en la forma de pensar, sentir e interactuar con los demás y con uno mismo», mencionaron la incorporación de prácticas caritativas a través del apoyo emocional y material a los necesitados. Este hallazgo también concuerda con los datos cuantitativos, con puntuaciones altas en los factores «transformación de uno mismo y/o de la propia vida» y «ofrecer ayuda a otros», con una media de 3.52 y 3.46, respectivamente.

Otro hallazgo notable son las bajas puntuaciones de los factores positivos del SRCOPE relacionados con las instituciones religiosas, con una media de 2,35 para «acciones en busca de ayuda espiritual» y 3,19 para «búsqueda de apoyo del clero». Las experiencias descritas por los familiares en este estudio sugieren que el nacimiento de un niño provocó un cierto distanciamiento de las instituciones religiosas debido a las agotadoras exigencias que requiere el cuidado de un niño.

Según la literatura, los cuidados especializados, el alto nivel de vigilancia y la carga que supone el cuidado dan lugar a una restricción social de las familias de los niños que requieren cuidados continuos y complejos^(20,22)^. Además, muchos de estos niños son muy susceptibles a las infecciones, por lo que los familiares se ven obligados a permanecer en casa para evitar la exposición^(21)^. Este aislamiento social puede ser una estrategia de protección para que los familiares eviten comportamientos discriminatorios y el estigma hacia las discapacidades de los niños^(19)^. Según una metaanálisis cualitativo, la restricción social puede ser un intento de los padres de protegerse a sí mismos y a sus hijos de las normas de aceptación social establecidas en la sociedad^(22)^.

También es importante reconocer la relevancia de los estudios sobre religiosidad y espiritualidad en el contexto sociocultural de Brasil, un país considerado un rico mosaico tejido a partir de una diversa gama de tradiciones y prácticas que reflejan su compleja historia y diversidad cultural^(24)^. En el presente estudio predominaron los evangélicos, lo que ratifica los datos del último censo del Instituto Brasileño de Geografía y Estadística (en portugués, *Instituto Brasileiro de Geografia e Estatística*, IBGE)^(25)^, que indica que Brasil se ha convertido en un país predominantemente evangélico. No obstante, Brasil sigue caracterizándose por una pluralidad de creencias y religiones. Según los datos del censo del IBGE de 2022^(25)^, hay aproximadamente 579 700 instituciones religiosas en Brasil, lo que refleja la importante diversidad religiosa del país, que incluye instituciones católicas, evangélicas, afrobrasileñas, indígenas, islámicas, judías y budistas.

Si bien los resultados de este estudio son coherentes con la bibliografía existente, hay que reconocer algunas limitaciones. Una de ellas es el pequeño tamaño de la muestra, combinado con el uso de un método de muestreo por conveniencia no probabilístico, lo que aumenta el riesgo de sesgo de selección. Otra limitación se refiere a la falta de integración entre los datos cualitativos y cuantitativos en la sección de resultados.

## CONCLUSIÓN

Los familiares de este estudio utilizaron con mayor frecuencia estrategias de coping religioso-espirituales positivas, especialmente los de familias más pequeñas. Sin embargo, los familiares más jóvenes utilizaron con mayor frecuencia estrategias de coping religioso-espirituales negativas. En consonancia con estos hallazgos, los cuidadores revelaron una experiencia marcada por la confianza en los designios de Dios y el alivio del sufrimiento a través de la fe.

Los hallazgos de este estudio son coherentes con la bibliografía existente sobre la religiosidad y la espiritualidad entre los familiares de niños que requieren cuidados continuos y complejos. Estos resultados pueden servir de base para estrategias de intervención directa, al considerar las necesidades espirituales de estas familias como un elemento importante de sus necesidades generales de cuidados. Se recomienda que los profesionales de la salud, especialmente los enfermeros, adopten un nuevo paradigma de cuidados que tenga en cuenta las necesidades espirituales y religiosas de las familias a la hora de planificar sus intervenciones.

## Data Availability

Todo el conjunto de datos que apova los resultados de este estudo fue publicado en el próprio artículo.
